# Fourier-transform infrared anisotropy in cross and parallel sections of tendon and articular cartilage

**DOI:** 10.1186/1749-799X-3-48

**Published:** 2008-10-06

**Authors:** Nagarajan Ramakrishnan, Yang Xia, Aruna Bidthanapally

**Affiliations:** 1 Department of Physics and Center for Biomedical Research, Oakland University, Rochester, MI 48309, USA

## Abstract

**Background:**

Fourier Transform Infrared Imaging (FTIRI) is used to investigate the amide anisotropies at different surfaces of a three-dimensional cartilage or tendon block. With the change in the polarization state of the incident infrared light, the resulting anisotropic behavior of the tissue structure is described here.

**Methods:**

Thin sections (6 μm thick) were obtained from three different surfaces of the canine tissue blocks and imaged at 6.25 μm pixel resolution. For each section, infrared imaging experiments were repeated thirteen times with the identical parameters except a 15° increment of the analyzer's angle in the 0° – 180° angular space. The anisotropies of amide I and amide II components were studied in order to probe the orientation of the collagen fibrils at different tissue surfaces.

**Results:**

For tendon, the anisotropy of amide I and amide II components in parallel sections is comparable to that of regular sections; and tendon's cross sections show distinct, but weak anisotropic behavior for both the amide components. For articular cartilage, parallel sections in the superficial zone have the expected infrared anisotropy that is consistent with that of regular sections. The parallel sections in the radial zone, however, have a nearly isotropic amide II absorption and a distinct amide I anisotropy.

**Conclusion:**

From the inconsistency in amide anisotropy between superficial to radial zone in parallel section results, a schematic model is used to explain the origins of these amide anisotropies in cartilage and tendon.

## Background

**Figure 1:**
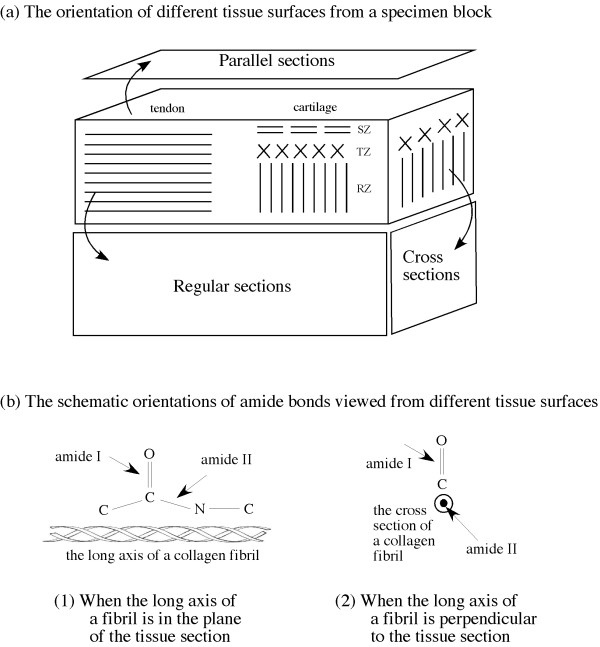
**The orientation of the different tissue sections from a specimen block (a) and the schematic illustration of the amide bonds at different tissue surfaces (b).** (SZ – superficial zone, TZ – transitional zone. RZ – radial zone).

Tendon is a soft connective tissue that lies in between bones and muscles in animal and human body to transfer the force experienced by muscle to the bone. Tendon therefore has the nature to resist mechanical tension. Depending upon the joint where it is placed, tendon can have different anatomic shapes [1]. Investigation on tendon has been carried out in various aspects [2-6] such as understanding the shape, structure, mechanical properties, tissue repair and structure-function relationship. Like tendon, articular cartilage is also a soft connective tissue, which covers the end surfaces of bones in synovial joints to distribute compressive loading. While type I collagen fibrils are commonly found in tendon as the highly organized and uniform fiber bundles, type II collagen fibrils are found maximally in articular cartilage that are organized in a depth-dependent structure [7-11], where the orientation of the local fibrils divides the cartilage depth into three sub-tissue zones, namely superficial (fibrils parallel to tissue's surface), transitional (random fibril orientation) and radial zones (fibrils perpendicular to the surface) (Figure 1a).

Structural and biochemical alteration in the microstructure and composition of molecular networks in the tissue due to any damage/degradation will eventually produce the clinical symptoms of osteoarthritis. Till date, osteoarthritis cannot be diagnosed at its earliest stage before the appearance of any clinical symptom. Change in biochemical constituents indicates the tissue degradation in advance. Recent studies of cartilage using Fourier-Transform Infrared Imaging (FTIRI) [12-19] show that infrared techniques with imaging capability have the potential to provide quantitative information about chemical composition of tissue in its native and degraded states. Since tendon has well-organized collagen structure, studies using infrared technique have also been initiated on tendon [20-22]. Following the earlier research on FTIRI of tendon and cartilage [20], efforts have been made to understand the infrared anisotropy of articular cartilage [23,24] for the tissue sections that consist of all the three zones (termed the regular section in this article, Figure 1a). In these anisotropy studies, multiple infrared imaging data were acquired, each for a different infrared polarization state in the 0°-180° angular space. Subsequent analysis using all images can provide detailed information regarding the fibril orientation and bond directions in cartilage [23,24]. When the long axis of the fibrils is in the plane of the tissue section, the bond directions of amide I and amide II are approximately perpendicular and parallel to the fibril axis respectively (Figure 1b1).

Since the matrix of collagen fibrils in articular cartilage has a unique three-dimensional (3D) structure, different surfaces of a tissue block should have the fibrils in different orientations, as illustrated in Figure 1a. In particular, we are interested in the infrared anisotropy when the long axis of the collagen fibrils is perpendicular to the plane of a tissue section. In such a case, a simple interpretation of the bond directions illustrated in Figure 1b1 would suggest a 'dot' for the amide II bond direction (Figure 1b2). In this study, the anisotropy of tendon and cartilage from all different surfaces of the 3D tissue cube were investigated using infrared imaging. To the best of our knowledge, there has been no study in literature regarding the infrared anisotropy in the regular/parallel/cross sections of tendon and cartilage. Since the infrared absorption of the amide I and amide II bonds has been found to have distinct anisotropy in articular cartilage [23,24], this article focuses on the features of these two amide components in all sections. (Since amide II and amide III bond directions are parallel, their anisotropy profiles are of similar pattern, whereas the sugar component of proteoglycan has no anisotropy [23].)

## Methods

### Sample preparation

Tendon and humeral head from mature canine, sacrificed for unrelated experiments, were used in this study. Fresh canine achilles tendon was cleaned and freed of fat, muscle and sheaths. Unfixed fragments of 10 mm long and 6 mm thick were cut, embedded in OCT compound (cryo-embedding medium) and snap frozen using liquid nitrogen. Special care was exercised in orienting the specimen parallel to the longest axis of the tendon. The specimen blocks were sealed in aluminum foil and stored at -80°C until use. Rectangular blocks of full thickness of cartilage attached to the underlying bone were harvested from the central load-bearing region of the humeral head. To monitor the influence of topographical variations, special attention was paid to the cartilage's location and orientation on the joint surface by preserving the interface between the soft tissue and the bone. The cartilage tissue blocks were placed in phosphate buffered saline (pH 7.3) to prevent drying and were refrigerated until use. Standard histology procedures were used to treat the cartilage tissue blocks, including overnight chemical fixation with formol-cetylpyridiniumchloride (CPC), decalcification with 10% ethylene diamine tetra acetic acid (EDTA)/Tris buffer for 7–10 days, and paraffin embedding in tissue processor (RMC PTP 1530). (The infrared spectrum of paraffin does not interfere with the cartilage spectra.)

Using a microtome (Micron HM325, Thermo Fisher Scientific, Waltham, MA), thin sections (~6 μm thick) were cut from different surfaces of tendons as well as cartilage tissue blocks and named as the regular, cross and parallel sections (Figure 1a). For articular cartilage, the regular sections contain all three zones of the tissue, with the long axis of the fibrils in the superficial and radial zones in the plane of the tissue section. The parallel sections of articular cartilage were acquired at different tissue depths. Hence, while the parallel sections from the superficial zone have the fibril in the plane of the sections, the parallel sections from the radial zone have the long axis of the fibrils perpendicular to the plane of the sections (cf Figure 1). Preserving the relative orientations among all parallel sections of cartilage is also critically important. For tendon, the long axis of the specimen is parallel to the long axis of the block; consequently, the regular and parallel sections of tendon have the fibrils running parallel in the plane of the sections. The cross sections of the tendon, however, only contain the 'ends' of the fibrils that are cut across, similar to the case of cartilage's parallel sections from the radial zone. These sections were placed on barium fluoride (BaF_2_) window as well as on commercially available mid infrared reflection study substrates called MirrIR slides (Kevley Technologies, Chesterland, Ohio) to conduct FTIRI experiments.

### Instrumentation details

Infrared images were acquired using a Spotlight 300 infrared imager from PerkinElmer (Wellesley, Massachusetts). The apparatus consists of a FTIR spectrophotometer and an infrared microscope. Liquid nitrogen cooled sixteen-element MCT (Mercuric Cadmium Telluride) detector with a moving stage for scanning the sample constitutes the microscope. The microscope also has a visible light source to focus the sample and to choose the region of interest for data acquisition. The sections fixed on the mechanical stage were undisturbed over the entire period of data collection. Experimental parameters were unaltered for the entire set of experiments.

To investigate the anisotropy, a commercial wire grid infrared polarizer from PerkinElmer was inserted between the sample and the detector (and will be referred as "*analyzer*" from now onwards). For each tissue section, infrared imaging experiments were repeated thirteen times with the identical parameters except a 15° increment of the analyzer's angle in the 0° – 180° angular space. For the analyzer angle 0°, the long axis of the collagen fibrils is parallel to the x-axis of the x-y moving stage of the Infrared Imager [23]. Transmission and reflection experiments were carried out for a selected region of interest on each tissue section with a pixel size of 6.25 μm^2^. The spectral resolution of the instrument is 16 cm^-1^with data interval 8 cm^-1 ^and 2 scans per pixel. Two to three identical sections were investigated in each type of experiment; the results were highly consistent. Other experimental details can be found elsewhere [23,24].

### Data analysis

Each single infrared imaging experiment produces a 3D data cube, two spatial dimensions and one spectral dimension in the mid infrared region (4000-750 cm^-1^). From this data cube, it is possible to extract two-dimensional (2D) chemi-maps for any desired spectral interval. It is also possible to examine the infrared spectrum at any spatial location of the tissue section. Since the previous studies have established the anisotropy profile for amide I and amide II components of articular cartilage in the spectral range 2000-1000 cm^-1^, this investigation also explored this spectral region. The baseline corrected chemi-maps were extracted for amide I and amide II from the spectral range 1700 to 1600 cm^-1 ^and 1600 to 1500 cm^-1 ^respectively, from all infrared images. In the case of tendon, eight by eight pixels in the chemi-maps were averaged to analyze the anisotropy at different surfaces of the block for both amide I and amide II. Similar averaging was done for the parallel sections of cartilage. For the regular sections of cartilage, eight consecutive columns were averaged into one full-depth column so as to preserve the depth resolution of the cartilage at 6.25 μm. Identical experimental parameters and data analysis approach were used for all tissue sections from three different specimens, which in turn yielded consistent results.

## Results

**Figure 2:**
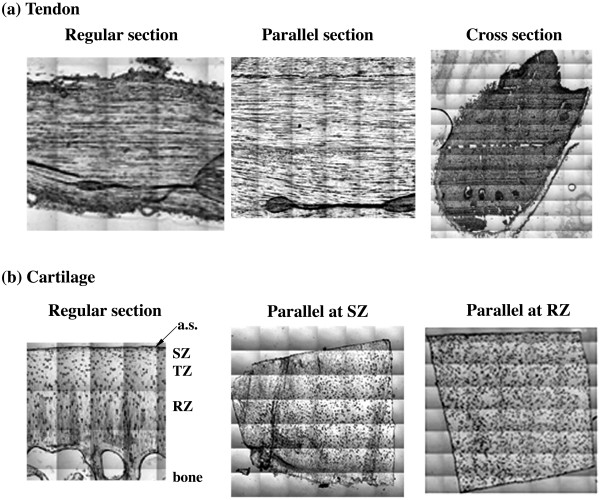
**The visible images from the FTIR imager, tendon (a) and cartilage (b).** (a.s. – articular surface).

Figure 2 shows the visible images of tendon and cartilage sections from different surfaces of the tissue block. It is evident that the regular and parallel sections of tendon have similar fibril morphology, with the tendon fibrils running parallel in the plane of the tissue section. In contrast, the cross section of tendon has very different morphology. For articular cartilage, the regular section contains three typical histological zones; whereas each parallel section of cartilage has a very different fibril orientation, depending upon the depth at which the section is obtained.

### Tendon results

**Figure 3:**
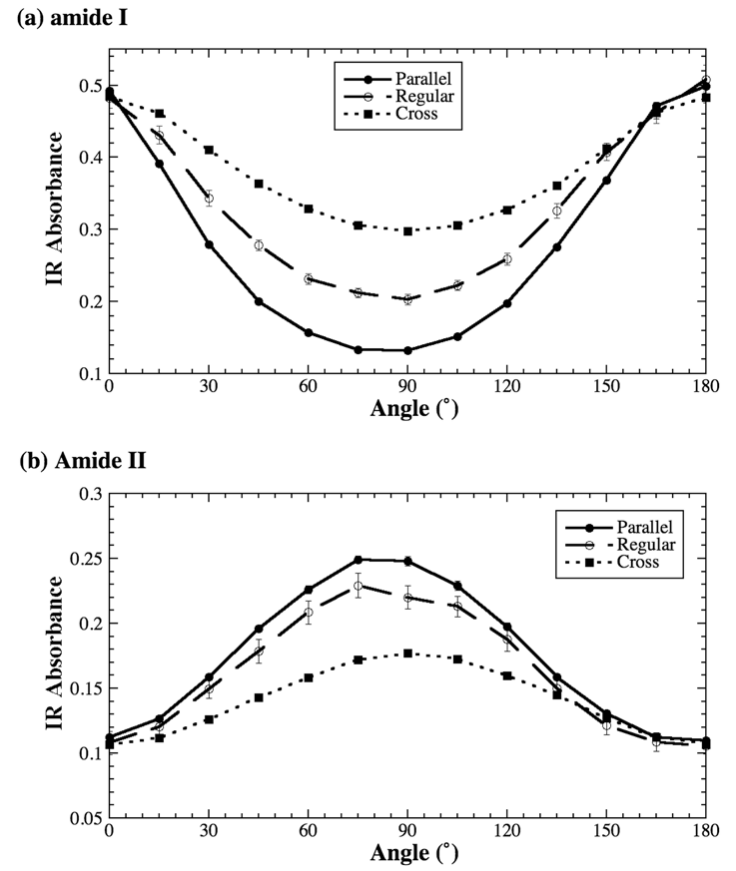
Absorption anisotropy of amide I (a) and amide II (b) of tendon in the regular, parallel and cross sections.

Figure 3 depicts the absorption anisotropy of amide I and amide II in tendon's regular, parallel and cross sections. Two features can be observed. First, the absorption anisotropy of amide I is stronger than that of amide II, which is due to greater bond strength (double bond) of amide I whereas amide II absorption is caused by lesser bond strength (single bond). Second, the anisotropy of amide I absorption is opposite to that of amide II for all three sections, that ensures the perpendicularity of transition moment directions of these amide bonds. For the parallel and regular sections of tendon, since the fibril's long axis is parallel to the x-axis of the moving stage in both orientations, their infrared anisotropy is similar to that of the radial zone fibrils in regular sections of articular cartilage (the amide I anisotropy has a maximum at 0° and a minimum at 90°; and the same for amide II is opposite [23,24]). An interesting result is the amide anisotropy in the cross sections – though the anisotropy is weaker compared to the same in other two surfaces, the angular dependency remains the same.

**Figure 4:**
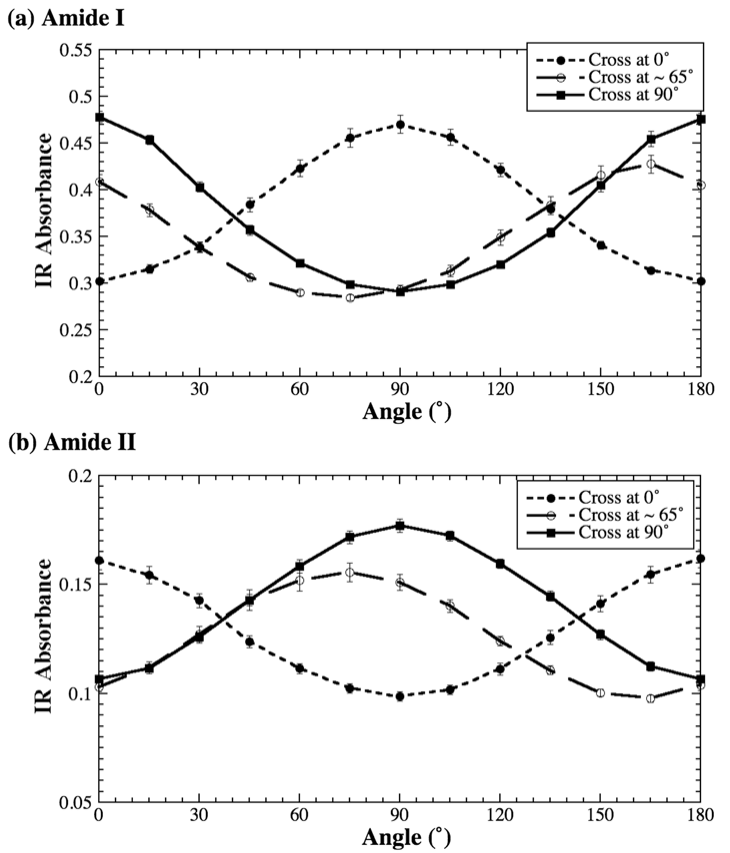
Absorption anisotropy of amide I (a) and amide II (b) of tendon's cross section at three different sample orientations.

To further investigate the anisotropy of the cross sections from tendon, the same cross section was placed at three different orientations (θ = 0°, ~65° and 90°) with respect to the polarization axis and the anisotropy experiments were repeated at these three orientations. Figure 4 shows the anisotropy profiles of amide I and amide II for these three orientations. It is clear that both amide vibrations have distinct anisotropy with the perpendicularity between them, even though the cross sections of the tendon do not have a visible fibril arrangement (cf Figure 2a). This result has two implications. First, the schematic assumption for the amide II orientation as illustrated in Figure 1b2 needs further investigation (see later in Discussion). Second, these amide bonds have a fixed orientation in the tissue's cross section with respect to the local fibril structure. (These experiments were conducted on various cross sections and the results are found to be consistent.)

**Figure 5:**
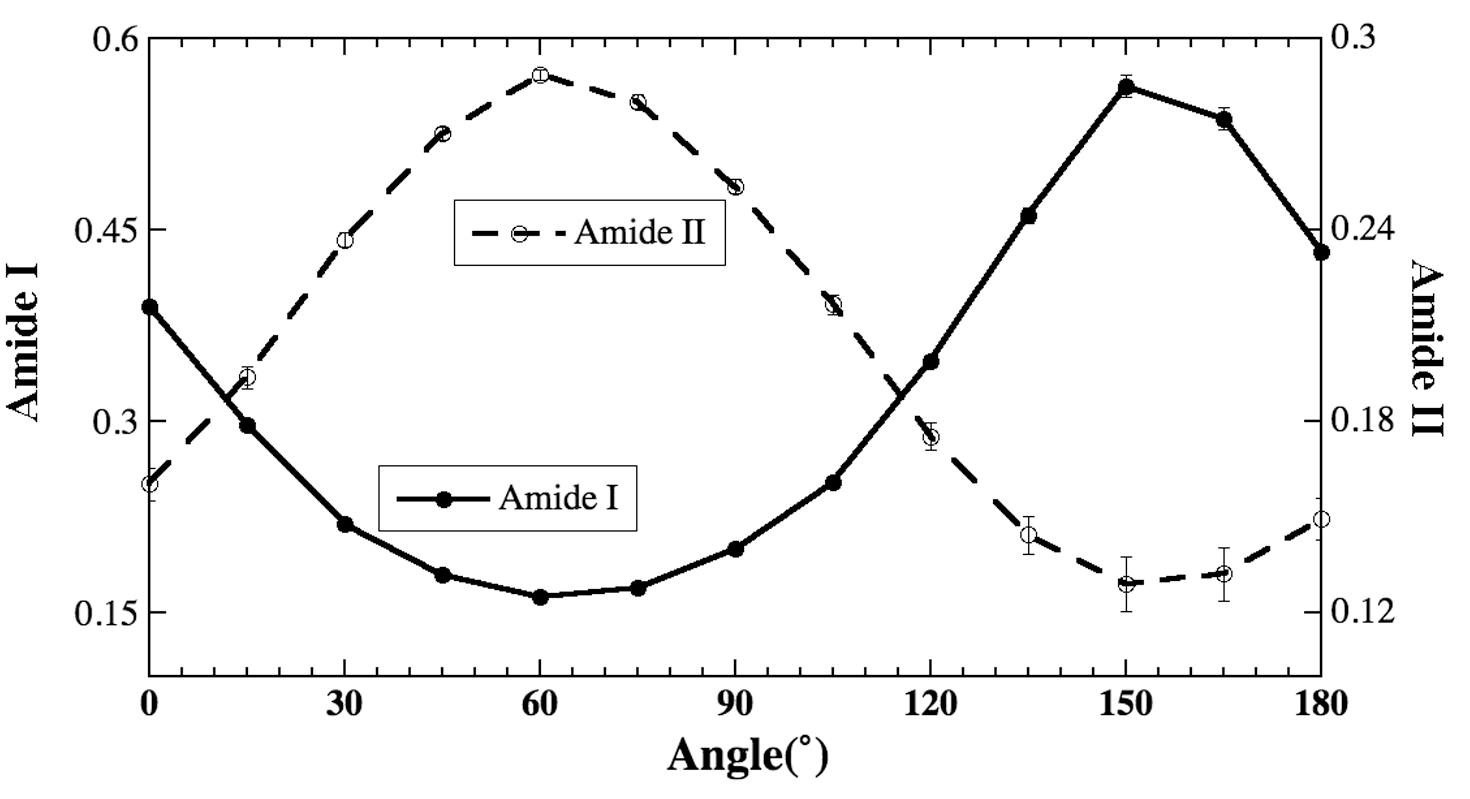
The phase shift in the absorption anisotropy due to a sample rotation (the same tendon section as in Figure 3 now oriented at ~60°).

Another noticeable feature in Figure 4 is the 'phase shift' in the minimum and maximum absorption locations (angles) for a sample that is not oriented parallel/perpendicular with respect to the analyzer 0°. Though it appears like a full cycle in 0–180° angular space, the difference between the minimum and maximum absorption will always be 90°. To verify this observation, the regular section of the tendon was imaged when the section was tilted by about ~60° with respect to the initial orientation used in Figure 3. The results are shown in Figure 5, where both profiles of the amide anisotropy from this regular section show the 'phase shift'. (i.e., the amide I plot in Figure 5 is 'phase shifted' from the amide I plot in Figure 3a.) This anisotropy shift illustrates the importance of the specimen orientation in the FTIRI anisotropy experiment, as the anisotropy is a polarization dependent phenomenon.

### Cartilage results

Based on the results of tendon, investigations are made on the anisotropy of cartilage for both regular sections as well as the parallel sections obtained at different zones. The results of the regular sections (that contain all three histological zones) are identical to our previously published data [23,24]. Since the fibrils are in the plane of the cartilage's regular sections, which is similar to the fibril orientation of tendon's regular/parallel sections, the amide anisotropy in these cartilage sections is identical to those in the tendon's regular/parallel sections (cf Figure 3). The only additional feature in the cartilage case is the perpendicular nature the fibril orientation between the superficial and radial zones of the tissue (cf Figure 1a), which causes the infrared anisotropy of the same amide component to be opposite between the two zones.

**Figure 6:**
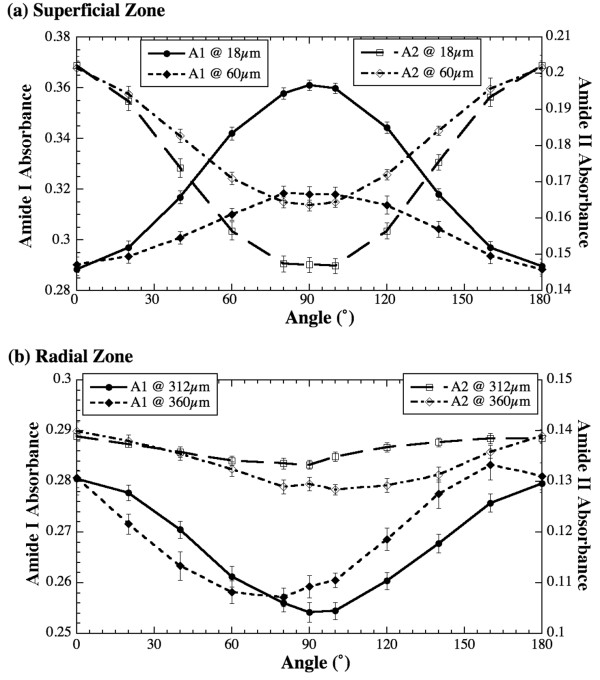
Representative infrared anisotropy profiles of amide profiles at the superficial zone (a) and the radial zone (b).

Since the regular section anisotropy is well-established, parallel sections of cartilage is focused in this article. Figure 6 shows the infrared anisotropy profiles at the superficial and radial zones of cartilage parallel sections respectively. In the superficial zone (Figure 6a), the anisotropy of amide I is opposite to that of amide II, which is the same as in regular section of cartilage. A unique feature of the infrared anisotropy in cartilage is its depth dependency. In regular sections of cartilage, the anisotropy of both amide components decreases gradually from the superficial zone to the transitional zone and increases in opposite direction gradually from the transitional zone to the radial zone. In this study where each parallel section has a 6-μm separation from the next one, the same trend in infrared anisotropy is observed. The two plots of amide I and amide II (Figure 6a) are from two parallel sections, separated by a 42 μm gap in between. The deeper section has the same but weaker anisotropy.

The parallel sections from the radial zone, however, have a different anisotropy. Figure 6b shows that, while amide I retains a distinct anisotropy, the amide II anisotropy in the radial zone becomes very weak. For this type of canine cartilage, the transitional zone has been found approximately from 70 μm to 120 μm [25]. From about 250 μm onwards, the tissue is well into its radial zone where all fibrils are expected to be parallel to each other (cf Figure 1a). Consequently, all parallel sections in the deep radial zone are expected to have the same anisotropy. This is true since there is little variation between the two plots of each amide component in Figure 6b, even the two parallel sections are 48 μm apart. However, the observation of amide I anisotropy in the radial zone's parallel sections was not expected, if one considers the schematic illustrations in Figure 1.

## Discussion

**Figure 7:**
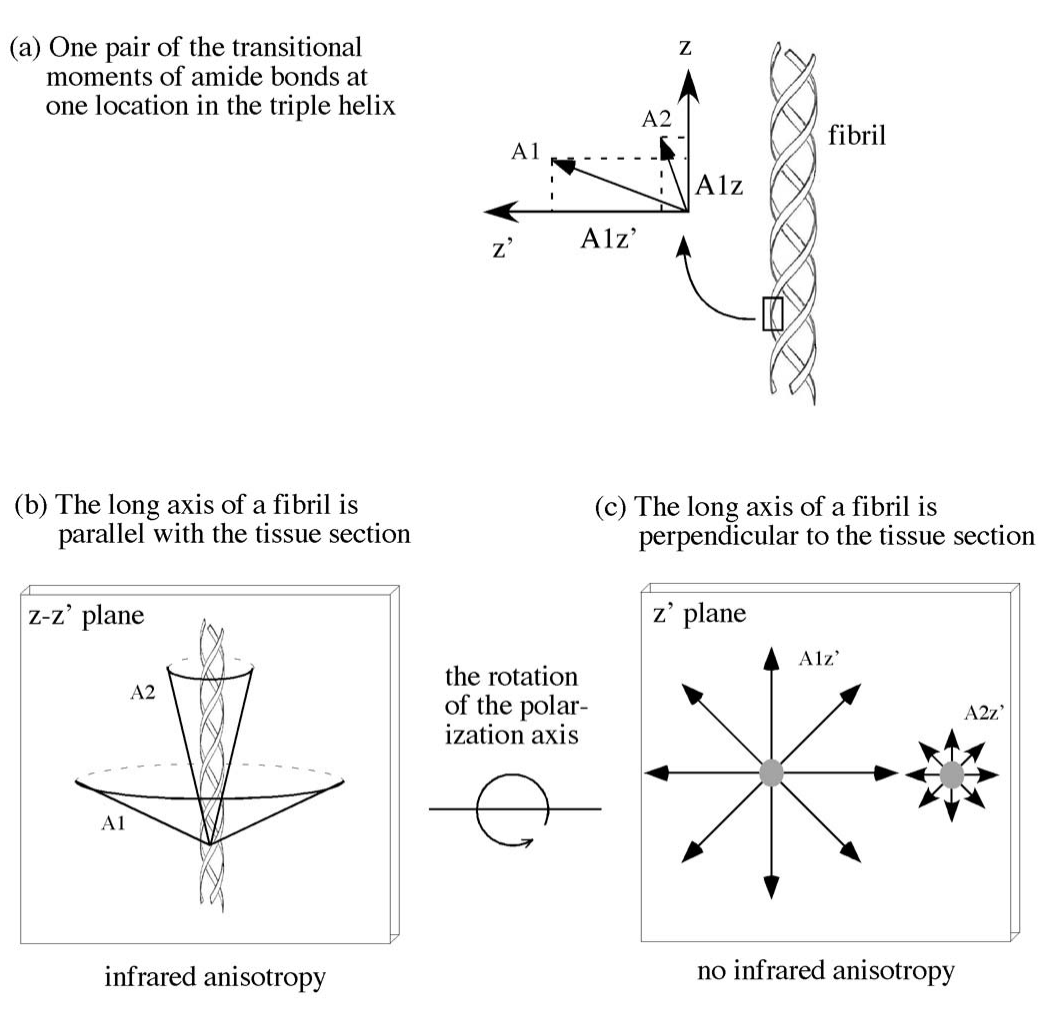
**(a) The transitional moments of one pair of amide bonds at one location in the triple helix.** The distribution of numerous amide bonds along the fibril axis would be similar to the cone structures as in (b). When the long axis of the fibrils is parallel to tissue section (b), the 'projection' of the transition moment 'cone' varies its size at the 2D z-z' plane with the change of polarization state. Consequently, there will be infrared anisotropy in (b). When the long axis of the fibrils is perpendicular to the tissue section (c), the 'projection' of the transition moment 'cone' remains the same at the 2D z' plane regardless of the polarization state. Consequently, there will be no infrared anisotropy in (c).

In infrared polarization experiments with cartilage/tendon, maximum and minimum absorption occurs when the polarization axis is parallel and perpendicular to amide bond transition moment directions respectively. Our previous results have verified such anisotropy for both amide I and amide II components using the regular sections of cartilage, as illustrated in Figure 1b1. To investigate infrared anisotropy for the tissue sections where the long axis of the fibrils is perpendicular to the section plane, such simple illustration is not sufficient. Hence, a detailed illustration is given in Figure 7, which incorporates the tilting angles of the transitional moments of amide bonds in collagen fibrils as well as the effect of polarization in infrared imaging.

It is well known in literature that the transition moments of amide I and amide II have tilting angles with respect to the axis of the alpha-helix [26], as shown in Figure 7a. Since the amide bonds are fixed in the peptide chains and the fibril contains three identical chains in a triple helix, it is evident that the transitional moments of amide vibrations also spiral around the long axis of the fibrils. Hence, there exist two situations when performing infrared polarization experiment: (a) when the long axis of the fibril is in the plane of the tissue section (Figure 7b), which is the case of regular/cross sections of cartilage with all three zones in the plane as well as the case of regular/parallel sections of tendon, and (b) when the long axis of the fibril is perpendicular to the plane of the tissue section (Figure 7c), which is the case of parallel section of cartilage in the radial zone as well as the cross sections of tendon.

When one carries out a polarization experiment, the polarization axis is *always*****rotated in the plane of the tissue section. In situation (a), the distribution of an amide bond around the numerous fibrils will have the shape of a cone, as shown in Figure 7b. When the polarization axis is rotated in the tissue plane (the z-z' plane), the transition moment vector of an amide component varies as the function of the polarization state, thus yielding anisotropy. In contrast, in situation (b), the infrared irradiation is polarized in the 2D z' plane, where the resultant bond vectors from the cone would not change as the function of the polarization state (Figure 7c), thus yielding no anisotropy.

In the study of tendon's cross sections, both amide I and amide II showed strong anisotropy (Figure 3). The result of cartilage's parallel sections from the radial zone, amide I has strong anisotropy but amide II is nearly isotropic (Figure 6b). The tendon results from the cross sections suggest that the long axis of the fibers is not perpendicular to the plane of the tissue section. This can be explained by a well known wavy/zigzag nature of collagen bundles in tendon [1,2], which may result in a residual anisotropy in tendon's cross sections observed in this study. In comparison, the isotropic nature of the amide II component in the parallel sections of cartilage's radial zone implies that the long axis of the fibrils in the radial zone of the cartilage is indeed perpendicular to the plane of the tissue section (the situation (b) discussed above). However, the same situation (b) cannot explain the observed anisotropy of amide I in the radial zone of cartilage. This conundrum might be, in part, due to the small concentration of connecting fibrils in cartilage's radial zone [27]. Further experiments have been planned to investigate the nature of the 3D fibril orientation in articular cartilage.

## Conclusion

The infrared anisotropy of tendon as well as cartilage has been investigated in regular, parallel and cross sections from a 3D tissue block. The results are mutually consistent, when the long axis of the fibrils is parallel to the plane of the tissue section. An interesting situation is when the long axis of the fibrils is perpendicular to the plane of the tissue section. Though the infrared anisotropy of amide components in tendon cross sections would be expected to be isotropic, the experimental results show a clear anisotropy for both amide I and amide II components in tendon. This could be attributed to the zigzag nature of the collagen fibers in tendon. The results of the parallel sections from cartilage's superficial zone are similar to that of regular sections in cartilage, which is also comparable to the anisotropy in tendon's regular and parallel sections. The parallel sections from cartilage's radial zone have a nearly isotropic amide II absorption and a distinct amide I anisotropy. The origins of these features are investigated with the aid of a schematic model.

## Competing interests

The authors declare that they have no competing interests.

## Authors' contributions

NR carried out the FT-IR imaging experiments, analyzed the raw data and drafted the manuscript. YX conceived of the study, participated in its design and coordination, finalized the data analysis, interpreted the concluding results, made the final figures, revised and completed the manuscript. AB performed the histological sectioning of the tissue blocks and participated in the experiments. All authors read and approved the final manuscript.
